# The Usefulness of Prioritization of Ivabradine Before Beta-Blockers in a Heart Failure Patient Suffering From Intra-hemodialysis Hypotension

**DOI:** 10.7759/cureus.40609

**Published:** 2023-06-18

**Authors:** Satoshi Yamaguchi, Nokanan Nadoyama, Kazushi Kinjo, Nobumori Yagi, Hiroshi Ishimori, Michio Shimabukuro

**Affiliations:** 1 Department of Diabetes, Endocrinology and Metabolism, School of Medicine, Fukushima Medical University, Fukushima, JPN; 2 Department of Cardiology, Nakagami Hospital, Okinawa, JPN; 3 Department of Nephrology, Nakagami Hospital, Okinawa, JPN

**Keywords:** beta-blockers, hypotension, hemodialysis, heart rate, dialysis, heart failure, beta blocker, ivabradine

## Abstract

Depressed cardiac systolic function in hemodialysis patients occurs for a variety of reasons and is a clinical problem. Beta-blockers are a key drug in the treatment of heart failure; however, hypotension may occur, particularly in dialysis patients, thereby complicating dialysis. Ivabradine has the unique property of a negative chronotropic effect only, without the negative inotropic effect. A 55-year-old woman who underwent dialysis presented with dyspnea and fatigue even at rest due to low cardiac systolic function. The left ventricular ejection fraction (LVEF) was 30%. Medications for heart failure, such as carvedilol and enalapril, were initiated; however, they were discontinued owing to intradialytic hypotension. Subsequently, her heart rate increased to over 100 beats per minute (bpm); therefore, we administered 2.5 mg of ivabradine before beta-blockers, which reduced her heart rate by approximately 30 bpm without a significant blood pressure decrease. Moreover, her blood pressure stabilized during dialysis. After two weeks, we added 1.25 mg of bisoprolol and adjusted the dose to 0.625 mg. After seven months of treatment with 2.5 mg ivabradine and 0.625 mg bisoprolol, systolic cardiac function significantly improved to 70% of LVEF. Prioritizing ivabradine over beta-blockers may not cause intradialytic hypotension; even low doses of ivabradine and bisoprolol were considered effective heart failure therapies.

## Introduction

Ivabradine is a selective hyperpolarization-activated cyclic nucleotide-gated channel antagonist that reduces the heart rate in sinus rhythm without a negative inotropic effect [1]. Clinical data suggest that ivabradine has a more negligible effect on blood pressure than beta-blockers [2].

Impaired cardiac systolic function is common in patients undergoing dialysis, which is multifactorial, including ischemic insults [3,4]. Nevertheless, in patients undergoing dialysis, beta-blockers play a pivotal role in the treatment of heart failure with reduced ejection fraction (HFrEF) [5]. The difficulty in initiating beta-blockers due to intradialytic hypotension is a potential clinical problem. Heart rate control with ivabradine may allow for sufficient left ventricular diastolic time, resulting in favorable physiological myocardial performance [2]. Previous studies [6] have demonstrated that ivabradine improved hemodynamics with preserved blood pressure in non-dialytic patients. Here, we report a case in which low-dose ivabradine dramatically improved cardiac contractility and stabilized blood pressure in a patient undergoing dialysis.

## Case presentation

**Figure 1:**
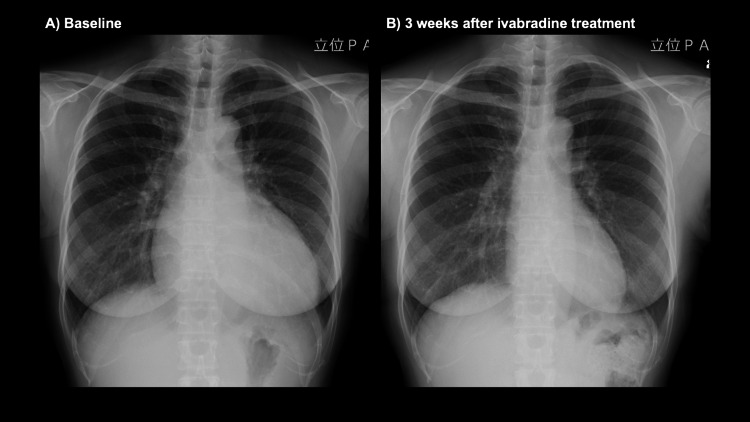
Chest X-ray before and after ivabradine treatment (A) Baseline. The chest-thoracic ratio was 58%. (B) Three weeks after ivabradine treatment. The chest-thoracic ratio was 42%.

A 55-year-old woman underwent dialysis three times a week for seven years because of non-diabetic renal disease. She visited our clinic because of shortness of breath and fatigue even while resting. Transthoracic echocardiography at dialysis initiation showed diffusely reduced left ventricular wall motion with a left ventricular ejection fraction (LVEF) of 44%. The LVEF was 44%, and the heart rate was sinus tachycardia (>100 beats per minute). Over the past six years, she developed heart failure. Recently, she gradually became concerned about breathlessness during exertion, such as walking stairs without chest pain. Finally, she felt fatigued even at rest. During scheduled dialysis, blood pressure often decreases. Nevertheless, a chest radiograph showed a large chest-thoracic ratio indicating over-volume (Figure 1A), and dry weight could not be adjusted because of intra-dialysis hypotension.

**Figure 2:**
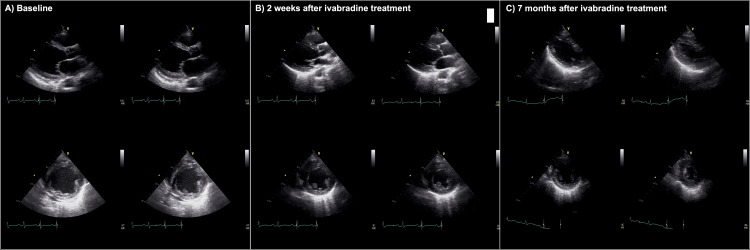
Transthoracic echocardiography before and after ivabradine The upper row presents the parasternal long axis view, and the lower row presents the parasternal short axis view. (A) Baseline. (B) Two weeks following ivabradine treatment. (C) Seven months following ivabradine treatment.

Eventually, a reduced cardiac systolic function was discovered on routine transthoracic echocardiography. The LVEF dropped to 30% (Figure 2A and Videos 1, 2).

**Video 1:** Transthoracic echocardiography before and after ivabradine The video shows the time course of transthoracic echocardiography.

**Video 2:** Transthoracic echocardiography at baseline Transthoracic echocardiography at baseline from parasternal long axis view, parasternal short axis view, apical four-chamber view, apical two-chamber view, and apical three-chamber view.

**Figure 3:**
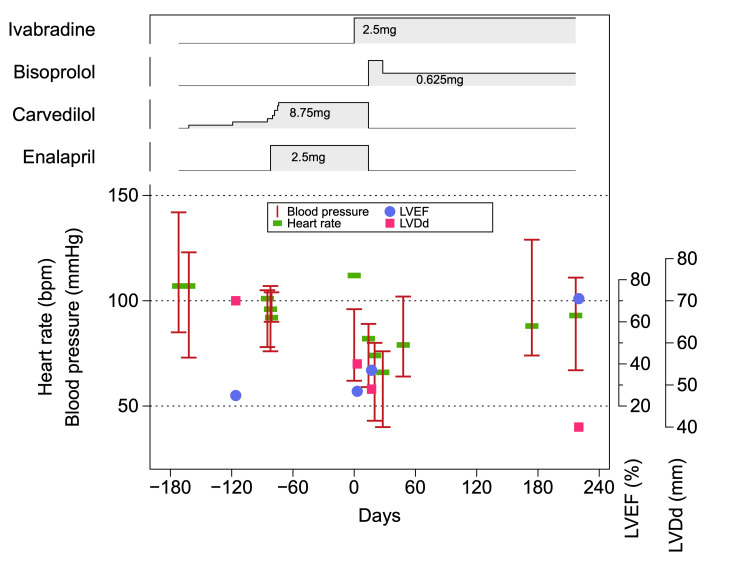
Clinical course Day 0 was set as the day ivabradine was started. LVDd, left ventricular dimension at diastole; LVEF, left ventricular ejection fraction.

She visited our clinic. We confirmed that there was no significant organic coronary artery stenosis on the coronary angiography. Carvedilol was started at 2.5 mg and titrated up to 8.75 mg over three months, and enalapril 2.5 mg was started simultaneously (Figure 3).

Carvedilol and enalapril were administered every morning before hemodialysis. Intradialytic hypotension developed after the initiation of these drugs. She experienced presyncope symptoms and collapsed several times because of orthostatic hypotension. Both carvedilol and enalapril were immediately discontinued to treat the low blood pressure (96/62 mmHg) with sinus rhythm at rest. Subsequently, her heart rate increased to >100 beats/minute. Ivabradine 2.5 mg/day was administered once daily under the electrocardiac monitor during hospitalization. The heart rate decreased by approximately 30 bpm within two weeks without a significant decrease in blood pressure. Figure 2B shows transthoracic echocardiography after two weeks of ivabradine treatment (Videos 1, 3).

**Video 3:** Transthoracic echocardiography after two weeks of ivabradine treatment Transthoracic echocardiography after two weeks of ivabradine treatment from parasternal short axis view, apical four-chamber view, apical two-chamber view, and apical three-chamber view.

Moreover, the blood pressure stabilized during dialysis. After two weeks, we added 1.25 mg of bisoprolol, and the dose was adjusted to 0.625 mg. Thereafter, these drugs were continued at the same dosage, and hypotension during dialysis was no longer observed.

Heart rhythms were monitored during hemodialysis. The cardiac rhythm was preserved in sinus rhythm, and atrial fibrillation was not observed. The heart rate dropped to the 70s within two weeks of initiating ivabradine administration. Chest radiography showed chest-thoracic ratio reduction (Figure 1B). Thereafter, it remained constant in the sinus rhythm. Seven months after ivabradine initiation, the systolic cardiac function significantly improved. The LVEF reached 71% on transthoracic echocardiography, and the left ventricular size also significantly decreased (Figure 2C and Videos 1, 4). She realized an improvement in shortness of breath while moving.

**Video 4:** Transthoracic echocardiography after seven months of ivabradine treatment Transthoracic echocardiography after seven months of ivabradine treatment from parasternal long axis view, parasternal short axis view, parasternal three-chamber view, and apical four-chamber view.

## Discussion

Ivabradine, the so-called pure heart rate drug, decreases heart rate without adverse inotropic effects [1]. Therapeutic drugs for heart failure, such as angiotensin-converting enzyme inhibitors or beta-blockers, generally depress blood pressure [7]. Despite the undeniable cardioprotective effects of beta-blockers in patients undergoing dialysis with reduced cardiac systolic function, intradialytic hypotension sometimes limits their use. Heart rate control with ivabradine (2.5 mg) and bisoprolol (0.625 mg) might stabilize intradialytic hypotension, which may allow appropriate plasma volume management, resulting in significantly improved LVEF and favorable reverse remodeling for only seven months (Figure 2 and Video 1).

Reduction in heart rate with ivabradine can improve physiological myocardial performance and increase stroke volume [8]. The SHIFT study, a randomized placebo-controlled study, investigated the clinical implications of heart rate-lowering therapy with ivabradine and showed that ivabradine can reduce the risk of cardiovascular death or hospital admission [9]. Ivabradine was also consistent with cardiovascular risk reduction in patients with normotensive end-stage renal disease patients [10]. However, there is little available evidence of the clinical efficacy of ivabradine in patients with low LVEF and intra-hemodialysis hypotension. Theoretically, ivabradine does not lower blood pressure, and some reports have demonstrated a neutral or increasing effect of ivabradine on blood pressure [6]. Ivabradine stabilized blood pressure within the first two weeks of administration in this case. Therefore, it might be advantageous to prioritize ivabradine over beta-blockers.

Aggressive up-titration of beta-blockers for the treatment of heart failure in patients undergoing dialysis has been debated [11]. In this case, only a small dose of beta-blockers and ivabradine was administered due to hypotension during dialysis; however, these drugs were sufficient to control the heart rate. Cice et al. [12] reported one-year beta-blocker reduced left ventricular volume and improved LVEF in end-stage renal disease patients with dilated left ventricle and low LVEF. Tachycardia with a low LVEF might worsen LVEF. Thus, ivabradine may play a key role in heart rate control. Adequate heart rate control might be effective in the treatment of heart failure without increasing the dose of beta-blockers. Ivabradine is mostly metabolized by the cytochrome P450 (CYP) enzyme CYP3A4, with less than 20% of renal metabolism [13]. Therefore, ivabradine can be safely initiated in patients with renal failure, and its clinical efficacy was achieved at a low dose in this case. Finally, improved LVEF may enable appropriate plasma volume management in patients undergoing hemodialysis.

The patient's cardiac function had been compromised since the initiation of dialysis; however, no organic stenosis was found on coronary angiography. The mechanisms underlying heart failure are not completely understood. In this case, the benefit of ivabradine may have been remarkable because the mechanism of heart failure was non-ischemic and the myocardium was viable.

## Conclusions

Considering blood pressure management in patients undergoing dialysis, ivabradine might be a first-line therapy prior to beta-blockers in patients undergoing dialysis with a very low ejection fraction. Prioritization of ivabradine can be a safe and feasible therapy in patients with low-systolic ejection fraction and low blood pressure.
